# Development of a Fluorescent Microfluidic Device Based on Antibody Microarray Read-Out for Therapeutic Drug Monitoring of Acenocoumarol

**DOI:** 10.3389/fbioe.2022.848501

**Published:** 2022-03-29

**Authors:** J.-Pablo Salvador, Thomas Brettschneider, Christian Dorrer, M.-Pilar Marco

**Affiliations:** ^1^ CIBER de Bioingeniería, Biomateriales y Nanomedicina (CIBER-BBN), Barcelona, Spain; ^2^ Nanobiotechnology for Diagnostics (Nb4D), Department of Surfactants and Nanobiotechnology, Institute for Advanced Chemistry of Catalonia (IQAC) of the Spanish Council for Scientific Research (CSIC), Barcelona, Spain; ^3^ Robert Bosch GmbH, Applied Research 1–Microsystem Technologies, Microstructuring and Assembly (CR/ARY2), Stuttgart, Germany

**Keywords:** microarray, antibody, acenocoumarol, microfluidic, fluorescence

## Abstract

The development of a proof-of-concept point-of-care (PoC) device for the determination of oral anticoagulants determination is presented. Acenocoumarol (ACL) is prescribed to prevent certain cardiovascular diseases related to the prevention of deep vein thrombosis, pulmonary embolism, myocardial infarction, and stroke. Oral anticoagualant treatment (OAT) represents a population of 2% under treatment which has expenditures about $ 144 million in 2011. The main drawback for OAT is the associated narrow therapeutic window and the unpredictable dose-response relationship, which is one of the main causes for visiting the emergency room at the hospitals. In a previous work, family antibodies were produced for the simultaneous detection of ACL and warfarin (W) depending on the area of application. It was developed in different formats, indirect and direct, either with similar detectabilities and both assays quantifying the oral anticoagulants with high accuracy and reproducibility. We present the implementation of the already developed immunochemical method to a point-of-care (PoC) device to assist on the patient compliance assessment programs. In order to achieve this goal, a first development was performed implementing ACL ELISA assay into a microarray format with fluorescent read-out. The assay was successfully implemented achieving a LOD of 1.23 nM of ACL directly measured in human plasma. Then, a fully integrated microfluidic system is developed which incorporates the specific immunoreagents for the detection of ACL. The immunoreagents were attached onto the glass slide in a microarray format. The system is automatic, rapid, sensitive, and disposable that could help clinicians monitor patients under OAT. According to the fluorescent label of the ACL binding, the chip can be easily read with a scanner. The microfluidic system performed good according to the robust and reproducible signals, and subsequently yielded an accurate result.

## Introduction

Acenocoumarol (ACL) is a coumarin derivative-like warfarin and phenprocoumon and is a vitamin K antagonist that inhibits the coagulation factors II, VII, IX, and X (Ansell et al., 2004). This drug is the most prescribed anticoagulant agent in continental Europe. In contrast, warfarin and phenprocoumon is mainly prescribed in the United Kingdom/United States and Italy, respectively. These kinds of drugs are prescribed in order to prevent some thrombolytic events or treat other diseases such as myocardial infarction, deep vein thrombosis, pulmonary embolism, or patients that have suffered a heart valve change (Hirsh et al., 1998). Acenocoumarol (ACL) is one of the most used drugs for oral anticoagulant treatment (OAT), which is prescribed to prevent certain cardiovascular diseases related to the prevention of deep vein thrombosis, pulmonary embolism, myocardial infarction, and stroke. In deficiency of these drugs, the patient can be affected by clots, and in case of overdosing the patient can suffer indiscriminate hemorrhages. Also, there is a large inter and intraindividual variability depending on the genetic isoforms, but also bioavailability is affected by other factors such as age, diet, medications, life-style, or stress. Thus, depending on the patient, the dose can vary widely from 1 to 9 mg/day (Ufer, 2005), accordingly the INR values has to be measured in centralized laboratories. OAT represents about 2% of the total population under this treatment. In the United States, warfarin expenditures were about $ 144 million in 2011 (Kirley et al., 2012). Unfortunately, incorrect medication and errors are quite frequent due to the great fraction of elderly patients taking such medication. In 2005, nearly 25% of the adverse drug reactions (ADRs) visiting the emergency room at the hospitals were due to OAT (Budnitz et al., 2011).

Assessment of patient compliance becomes very important in the case of OAT due to the aforementioned particularities of these drugs. Determination of oral anticoagulants in plasma and urine has mainly been performed by using chromatographic techniques coupled to mass spectrometry (De Vries and Völker, 1990; Maurer and Arlt, 1998; Vecchione et al., 2007). Some studies have been carried out to correlate INR index to the OAT concentration, but showing that there is no association or poor correlation between INR and the plasma concentration (Lombardi et al., 2003; Jeyalakshmi et al., 2018). However, there are still interest in providing analytical tools for the correct adjustment of the dose according to its low therapeutic window (Nieuwlaat et al., 2011), determining accidental intoxication with OAT(Brands et al., 2021), and also for pharmaceutical specimens analysis in order to ensure the quality of the prepared formulations (Chaudhari et al., 2020). Immunochemical techniques offer the possibility to develop simple and reliable bioanalytical methods on a variety of configurations (test-strip, microplate assays, biosensors, *etc*) depending on the analytical needs (Marco et al., 1995). The key element is the availability of antibodies specifically designed and produced to bind with the high affinity target analyte. Immunochemical assays that are very complex for implementation as a lateral flow test usually require multiple sample preparations and pipetting steps that have to be performed by trained personnel, adding significant costs and increasing turnaround times. Alternatively, sophisticated pipetting robots can be employed; however, these machines are only profitable in large centralized labs with very high throughput. In order to enable a widespread application of OAT monitoring, an automated point-of-care testing is highly desired, for example, at the bedside, at the GP’s office, or even at private households.

In a previous work, family antibodies were produced for the simultaneous detection of acenocoumarol and warfarin depending on the area of application (Salvador et al., 2018). It was developed in different formats, indirect and direct, either with similar detectabilities, and both assays quantifying the oral anticoagulants with high accuracy and reproducibility. With this scenario, we have focused our efforts on developing diagnostic tools to fast and efficiently monitor the plasmatic levels of coumarins. As a proof-of-concept, a device for helping clinicians to monitor patients under OAT is developed, merging the immunoreagent of ACL determination and an autonomous device. This microfluidic system is polymer-based and goes beyond the state-of-the-art in particular in two aspects: i) a microfluidic element is provided for storing the required reagents close to the chip; after filling of this “reservoir block”, the user can walk away for the duration of the assay, and ii) the precise positioning of liquids inside a microfluidic system is not trivial, usually requiring light barriers or other complicated fluid control schemes. In our work, a dedicated technology employing “displacement chambers” was developed for precisely positioning the aliquots of reagents above the detection area; the required electronic periphery could therefore be greatly simplified. Due to the fact that the ACL-antibody binding is monitored with a fluorescent label, the obtained signal can be easily read with a scanner. The system was characterized by analyzing the robustness and reproducibility of the acquired signal and subsequently yielded an accurate result.

## Materials and Methods

### Chemicals and Immunoreagents

The coating antigen and antibody used for the detection of ACL (A2BSA and As233, respectively) have been described before (Salvador et al., 2018). The immunoreagents used as a control (13BSA and As138) are available in our laboratory and will be published elsewhere. The preparation of the bioconjugate competitors and the production of the antibodies have been performed with the support of the U2 of the ICTS “NANBIOSIS”, more specifically by the custom antibody service (CAbS, CIBER-BBN, IQAC-CSIC). The anti-rabbit IgG-TRITC was purchased from Sigma-Aldrich. Other chemical reagents were purchased from Aldrich Chemical Co. (Milwaukee, WII, United States). Acenocoumarol was purchased from Ipochem (Warszawa, Poland). Pooled human CPD plasma was purchased from 3H Biomedical AB (Uppsala, Sweden). Other chemical reagents were purchased from Aldrich Chemical Co. (Milwaukee, WI).

### Buffers

PBS is 10 mM phosphate buffer and 0.8% saline solution, and unless otherwise indicated the pH is 7.5. PBST is PBS with 0.05% Tween 20.

### Preparation of GPTMS Modified Slides

The slides (corning, 75 × 25 mm) were cleaned with piranha solution (H_2_SO_4_:H_2_O_2_, 7:3) for 1 h. The slides were then washed thoroughly with deionized water. Then, the slides were cleaned by immersing in a 10% (w/v) NaOH solution for 1 h at RT, followed by a surface functionalization using a 2.5% (v/v) 3-glycidoxypropyltrimethoxysilane (GPTMS) solution in anhydrous ethanol for 3 h at RT. The slides were dried under a stream of nitrogen, and then immersed into absolute alcohol for 30 min for final wash. The finished slides can be stored in a desiccation box at room temperature for several months.

### Microarray Printing

Haptenized BSA conjugates (13BSA and A2BSA, 25 and 50 μg/ml in PBS, respectively) were spotted onto GPTMS substrates using a BioOdissey Calligrapher MiniArrayer (Bio-Rad Laboratories, Inc. United States) in a humidity chamber at 60%, maintained for 1 h at 20°C, and stored in a desiccation box until use. Each glass slide contained 24-wells. A 2 × 5 spot matrix was printed on each well with five spots replicated for each haptenized BSA conjugates. For glass slide chip used for the PoC device, a matrix of a 10 × 3 spots was printed on each slide with 15 spots replicated for each haptenized BSA conjugates.

### Preparation of the Microarray (Format 24 Wells)

The assay was carried out placing the slides on a 96-well formatted microplate hardware (ArrayIt system, Sunnyvale, CA, United States) allowing experimentation with up to four glass substrate slides (Telechem International Inc.). The system was provided with a silicon gasket that demarcated into 24-wells per slide. Before starting the assay, the slides were washed four times with PBST. Once ready, ACL (0.128 nM–2 μM, a pooled human plasma) or the spiked human plasma samples were added to the wells (50 μL/well in human plasma) followed by the ‘‘As cocktail’’ (As233 and As138, at 1/250 and 1/8,000, respectively, 50 μL/well, PBST) and incubated for 30 min at RT. The slides were washed four times with PBST, and the aIgG-TRITC solution (1/250 in PBST, 100 μL/well) was added. After an incubation step of 30 min at RT, the slide was washed with water, dried with N2, and read with the scanner.

### Preparation of the Microarray (Format Chip)

Before starting the assay and connecting the microarray slides to the microfluidic chip, they were washed four times with PBST. Once ready, the ACL spiked/non-spiked plasma sample (100 and 0 nM, respectively, 50 μL) followed by the ‘‘As cocktail’’ (As233 and As138, at 1/250 and 1/8,000, respectively, 50 μL/well, PBST) was incubated for 30 min at RT. The slides were washed four times with PBST, and the anti-IgGTRITC solution (1/250 in PBST, 100 μL/well) was added. After an incubation step of 30 min at RT, the slide was washed with water, dried with N_2_, and read with the scanner.

### Data Evaluation

Measurements were recorded on a ScanArray Gx PLUS (Perkin Elmer, United States) with a Cy3 optical filter with 10-μm resolution. The laser power and PMT were set to 90 and 70%, respectively. The spots were measured by F543_Mean-B543 (Mean Cy3 foreground intensity minus mean Cy3 background intensity). Fluorescence intensity values were expressed normalized or in relative units as average and standard deviation of three replicate wells. The signal obtained from A2BSA/As233 assay was normalized with the data obtained from 13BSA/As138. The competitive curves were analyzed with a four-parameter logistic equation using the software GraphPad Prism v 4 (GraphPad Software Inc., San Diego, CA, United States). The standard curves were fitted to a four-parameter equation according to the following formula: Y = [(A-B)/1-(x/C)^D^] + B, where A is the maximal fluorescence, B is the minimum fluorescence, C is the concentration producing 50% of the difference between A and B (or IC50), and D is the slope at the inflection point of the sigmoid curve. The limit of detection (LOD) was defined as the concentration producing 90% of the maximal fluorescence (IC90).

### Fabrication of Microfluidic Device and Protocol

Microfluidic structures were milled from 1.5 mm thick injection-molded polycarbonate substrates (Makrolon 2,605, Bayer Material Science). Laser welding was employed to join substrates with an intermediate flexible and a light absorbing polymer membrane (Platilon U 4281 night black, Epurex Films GmbH). A fixed laser power of *p* = 1,500 mW in cw mode was employed. To cover channels running on the backside of the polymer stack, an adhesive tape (Polyolefin sealing foil, HJ Bioanalytik) was applied. For fluidic interfacing with PTFE and silicone tubes, metal tubular rivets were glued (Endfest 300, UHU GmbH) onto the microfluidic chips. For on-chip processing, glass slides containing the microarray were bonded against the microfluidic chip by a laser-structured double-side adhesive foil (170 μm Spacer Tape TV090844, Tesa SE). External pilot valves were controlled by a computer to apply atmospheric, positive or negative differential pressures (*p*_+_ = 60 kPa, *p*_−_ = −60 kPa) to the chip inlets. The throttles were set to yield displacement times of 10 s and washing intervals of 30 s. The reservoir block was sealed against the chip by O-rings. The adhesive tape was used to form an elongated detection cavity, allowing reproducible filling with reagents.

## Results and Discussion

Here, we present the development of a fluorescence antibody microarray for the detection of an oral anticoagulant such as ACL and the implementation in an automated microfluidic device. The aim of this work is to provide proof-of-principle for a diagnostic tool that allows an immunoassay in an easy and automatic way.

The election of the most suitable combination to implement in a fluorescent antibody microarray for ACL determination was performed based on the existing immunoreagents that were previously produced (Salvador et al., 2018). In our previous work, the best combination used was a direct format (hACL-HRP/As236) which reached an LOD of 1.23 nM in plasma. However, the combination A2BSA/As233 was chosen for the development of the fluorescent microarray due to this configuration allowed the easy fluorescent labeling required for the microarray reading. Thus, the indirect format was selected and will be implemented in the proposed platform. Also, combination A2BSA/As233 revealed a good detectability for warfarin in plasma (LOD of 1.5 nM) but also, a good cross-reactivity for ACL (84%). Both analytical features make this assay ideal for microarray development and, in this study, ACL was chosen as the standard.

**FIGURE 1:**
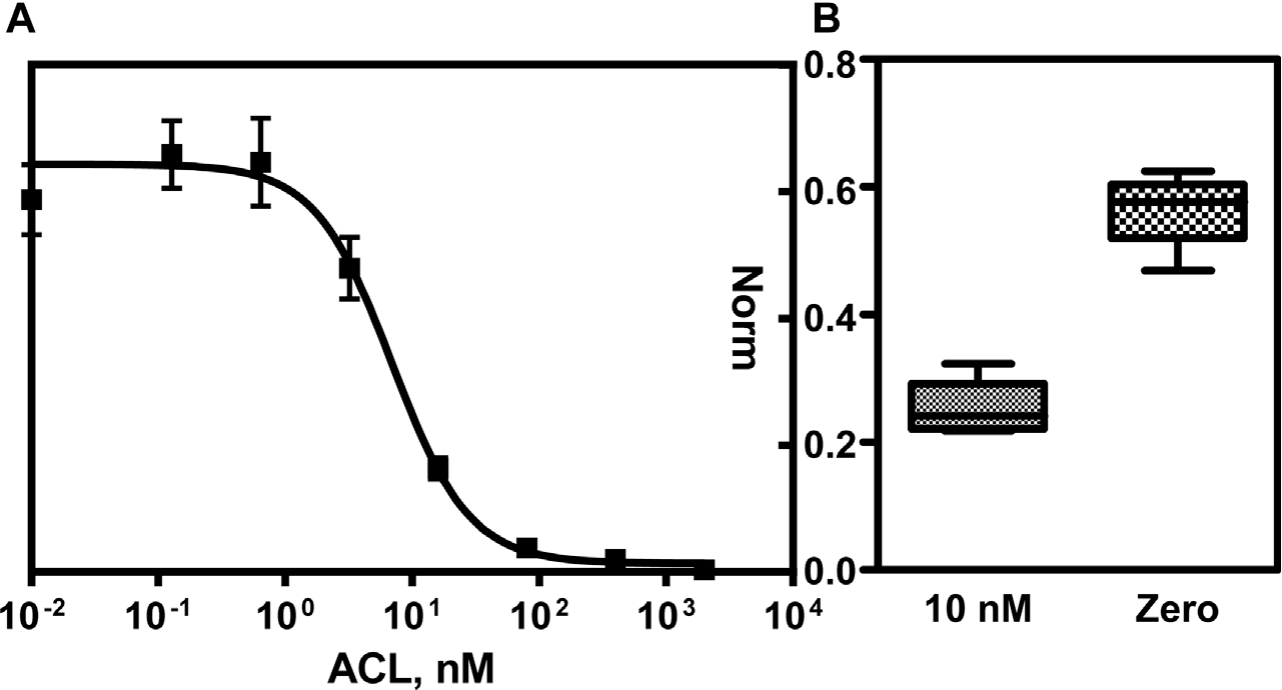
**(A)** Normalized calibration curve for A2BSA/As233 combination using as IS, 13BS/As138 assay. The SD corresponds to the mean value of eight assays performed in different days. **(B)** Box plot obtained from two samples (Zero: non-spiked and spiked at 10 nM in PBST) measured in separate wells. The SD corresponds to the mean value of four assays performed in different days.

Moreover, in order to improve the reproducibility intra/inter well, an internal standard (IS) was used. This IS will provide a constant signal that can be used to normalize the ACL binding assay or either, used as a control signal to ensure that the assay is working properly. The combination used was a couple of immunoreagents available in our laboratory, 13BSA/As138, which was used for the determination of anabolic androgenic steroids. For one side, the most suitable immunoreagent concentrations of A2BSA/As233 assay was performing a 2D checkerboard titration which consisted in testing different concentrations of the coating antigen A2BSA with different dilutions of As233. The optimum concentration was set in 50 ug/mL of A2BSA and As233 dilution factor in well of 1/500 in buffer. On the other hand, the concentration of 13BSA and As138 was set choosing the most suitable ones that reaches a good morphology of the spot and in the same range of the fluorescent signal that A2BSA/As233 maximum signal. According to these concentrations, a calibration curve was performed which was testing different amounts of ACL in buffer. Afterward, the signal was read and normalized using the IS signal (ratio A2BSA/As233 *vs*. 13BSA/As138). The calibration curve is showed in Figure 1A), and the analytical parameter are enclosed in Table 1. The detectability (LOD = 1.23 nM) achieved using the fluorescent microarray was similar compared to the previously obtained ELISA assay. According to the literature (Ufer, 2005) and with the data measured in the previous work (Salvador et al., 2018), the range of concentration found in plasma are within 20–200 nM. The working range obtained for the fluorescent antibody microarray would allow the measurement of ACL directly in plasma.

**TABLE 1:** Analytical features from the fluorescent microarray calibration curve for A2BSA/As233 assay using as IS 13BSA/As138 (N = 8). Quantification of sample at 10 nM (N = 4) is interpolated.

	Mean	SD	CV (%)
Norm_min	0.012	0.005	42
Norm_max	0.590	0.059	10
Slope	−1.466	0.228	16
IC_50_, nM	7.099	1.288	18
R^2^	0.995	0.005	1
LOD, nM	1.231	0.320	26
IC_80_, nM	2.351	0.600	26
IC_20_, nM	19.026	0.600	3
Sample (10 nM)	9.020	1.891	21

The device that planned to develop was thought with the aim to measure a single sample in a disposable way. Therefore, the reproducibility of the calibration curve has to be tested. As it can be observed in the Table 1, the calibration curve obtained was reproducible according to the CV estimated in 18% as a mean value. In order to prove the reproducibility of the assay measuring a specific concentration, a fortified sample was prepared at 10 nM of ACL and measured with an external calibration curve (see Figure 1B). The CV obtained was 21%, a CV similar to the calibrators, demonstrating that a sample quantified with an external calibrator can be performed. As it can be observed, non-significant difference was observed from zero samples, and the signal obtained at the maximum signal of the assay once interpolated the signal from the spiked sample at 10 nM in the calibration curve quantified 9.02 ± 1.89 nM.

**FIGURE 2:**
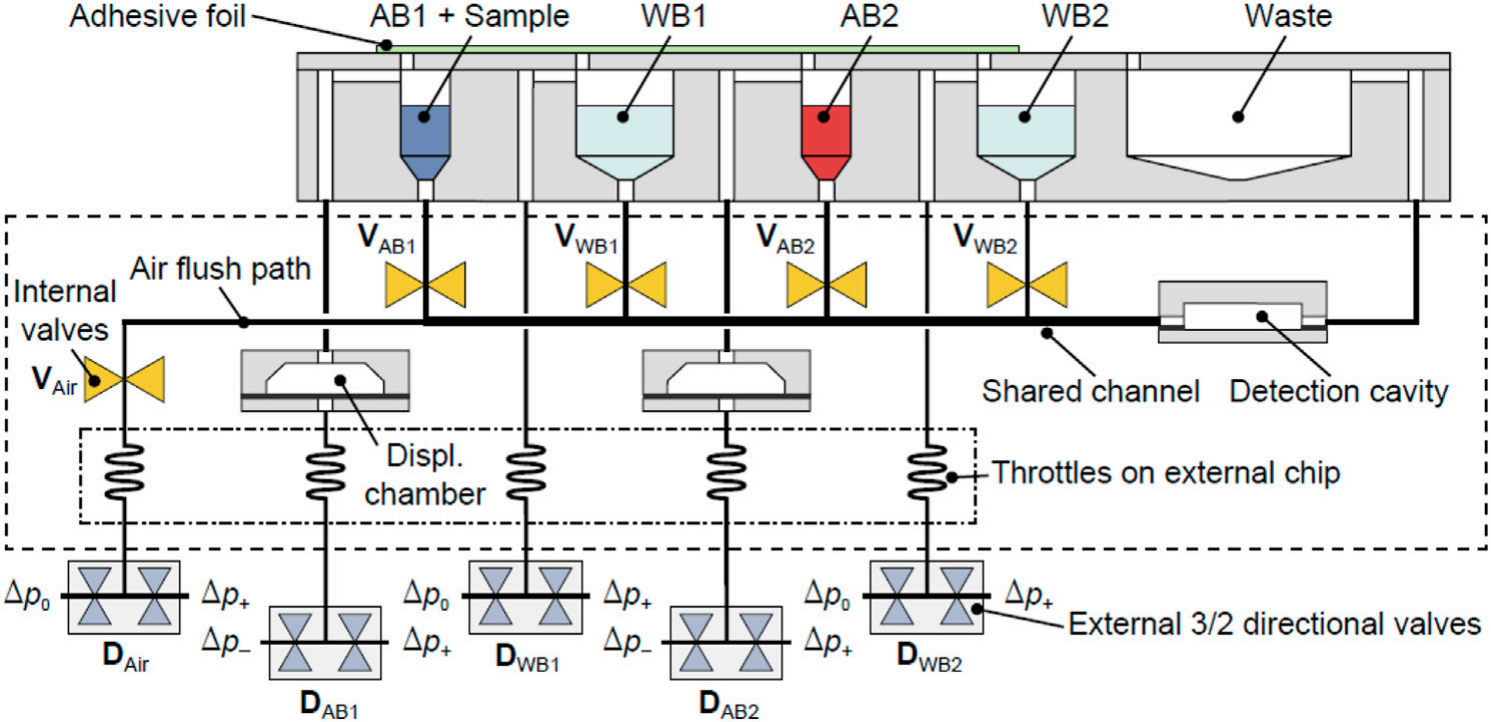
Schematic microfluidic layout employed to perform an immunoassay. Internal and external valves are labeled as V and D, respectively. Control valves to switch between positive (*p*+) and negative (*p*+) differential pressure and atmospheric pressure (p0) are indicated. AB1+sample reservoir content the sample plus the cocktail of antibodies used for the assay (As233 and As138) in their corresponding concentration in PBST. AB2 reservoir is used to contain the secondary antibody labeled with TRITC (aIgG-TRITC). WB1 and WB2 reservoir are filled with PBST.

To demonstrate the microfluidic approach, the assay was integrated using a set of different unit functions based on a pneumatically actuated microfluidic platform, which consists of two rigid polymer substrates separated by a flexible polymer membrane (Rupp, 2011; Brettschneider, 2014). Initially, the protocol of the assay had to be modifying in order to reduce the time of the assay and set up the volumes of the assay. The assay protocol is summarized in Table 2. It was decided to maintain the competition time among the three species (ACL, specific antibody, and coating antigen) in 30 min, while the revealing step with the fluorescent label anti-IgG was decided to reduce in 10 min. As it was described, this polymer membrane can be used to realize microfluidic valves, which are indicated by a circuit symbol in Figure 2. Antibody/sample solutions (AB1+sample, AB2) were placed in an external reservoir block to store them together with two wash reservoirs (WB1 and WB2) which provide the buffer for the washing steps and finally, a waste container for excess reagents. AB1+sample reservoir content the sample plus the cocktail of antibodies used for the assay (As233 and As138) in their corresponding concentration in PBST. AB2 reservoir is used to contain the secondary antibody labeled with TRITC (aIgG-TRITC). WB1 and WB2 reservoir are filled with PBST. All reservoirs were designed in separate cavities with dimensions large enough to collect the liquid by gravity at the bottom and, at the top of the cavity, a channel with a connection with the detection cavity. The reservoir block was connected to the microfluidic chip (outline indicated by dashed line), containing integrated membrane valves to prevent unwanted capillary flow from the reservoirs into the channel shared by AB1, AB2, WB1, and WB2 toward the detection cavity. Moreover, gas throttles, realized as narrow microfluidic channels in a separate “throttle chip”, were employed in the pneumatic lines leading to the valves and reservoir block to slow down actuation and enable gentle movement of fluids within the system.

**TABLE 2:** Assay protocol for the microfluidic device after holding the printed microarray slide.

Step	Time	Solution	Buffer	Volume	Temp
1	0′	Sample + As233/As138	Plasma + PBST	50 + 50 μL	RT
2	30′	Washing	PBST	3 × 100 μL	RT
3	31′	aIgG-TRITC	PBST	100 uL	RT
4	41′	Washing	PBST	3 × 100 μL	RT
5	42′	Drying	Air	—	RT
6	43′	Reading	dry	—	RT

Finally, besides a detection cavity including the microarray, displacement chambers were developed. This component made it possible to move a well-defined volume of liquid within the microfluidic system. Its operating principle is as follows: As shown in Figure 2, a displacement chamber consists of a recess in one of the polymer layers. The recess is covered by the flexible membrane, which can be deflected into the recess through the application of pneumatic pressure. As a result, a defined amount of gas is displaced.

The concept was based on the following sequence of steps (see Table 3). Initially, all internal valves (V) were closed, while the external directional control valves (D) were set to negative pressure *p*_−_ for the displacement chambers or atmospheric pressure p_0_ for all other inputs. Then, DAB1 was set to positive pressure *p*_+,_ and the valve VAB1 was opened, leading to the deflection of the polymer membrane into the first displacement chamber. A defined amount of gas was thereby displaced, in turn shifting a defined amount of antibody solution into the microfluidic system. Then, the antibody solution (AB1+sample) completely covered the detection cavity. The fact that a predefined displacement volume was set is that inherently this process is self-limiting, allowing to start incubation without further interaction. A metering unit formed by a combination of the displacement chamber and external reservoir allowed filling precisely through the detection cavity without the need for external pumps and active flow control, for example., by tracking the fluid meniscus with light barriers or CCD cameras, as is usually necessary in microfluidic systems where similar functions have to be performed.

**TABLE 3:** Table summarizing the sequence of assay steps and the corresponding conditions of internal valves (V) and external directional control valves (D) (c: closed, o: opened, valve abbreviations according to Figure 2). Entries without alteration to the preceding step were grayed out to increase readability.

Step	V_AB1_	V_WB1_	V_AB2_	V_WB2_	V_Air_	D_AB1_	D_WB1_	D_AB2_	D_WB2_	D_Air_
Initial	C	C	C	C	C	$\Delta p_{-}$	$\Delta p_{0}$	$\Delta p_{-}$	$\Delta p_{0}$	$\Delta p_{0}$
Displ. AB1	O	C	C	C	C	$\Delta p_{+}$	$\Delta p_{0}$	$\Delta p_{-}$	$\Delta p_{0}$	$\Delta p_{0}$
Air flush	C	C	C	C	O	$\Delta p_{+}$	$\Delta p_{0}$	$\Delta p_{-}$	$\Delta p_{0}$	$\Delta p_{+}$
Wash WB1	C	O	C	C	C	$\Delta p_{+}$	$\Delta p_{+}$	$\Delta p_{-}$	$\Delta p_{0}$	$\Delta p_{0}$
Air flush	C	C	C	C	O	$\Delta p_{+}$	$\Delta p_{0}$	$\Delta p_{-}$	$\Delta p_{0}$	$\Delta p_{+}$
Displ. AB2	C	C	O	C	C	$\Delta p_{+}$	$\Delta p_{0}$	$\Delta p_{+}$	$\Delta p_{0}$	$\Delta p_{0}$
Air flush	C	C	C	C	O	$\Delta p_{+}$	$\Delta p_{0}$	$\Delta p_{+}$	$\Delta p_{0}$	$\Delta p_{+}$
Wash WB2	C	C	C	O	C	$\Delta p_{+}$	$\Delta p_{0}$	$\Delta p_{+}$	$\Delta p_{+}$	$\Delta p_{0}$
Air flush	C	C	C	C	O	$\Delta p_{+}$	$\Delta p_{0}$	$\Delta p_{+}$	$\Delta p_{0}$	$\Delta p_{+}$

The washing step was then performed by setting the external valve DWB1 to positive pressure *p*_+_ and opening the valve VWB1, thereby flushing the wash buffer through the detection cavity until the reservoir was empty. This scheme was then repeated for the second antibody AB2 and wash buffer WB2. Additionally, after each binding step and washing time, the content of the detection cavity and common channels were removed using an air flush step to avoid cross-reaction between different immunoreagents and assured a well-defined state of the system prior to the next processing step. The final air flush step was optionally used to dry the detection cavity.

**FIGURE 3:**
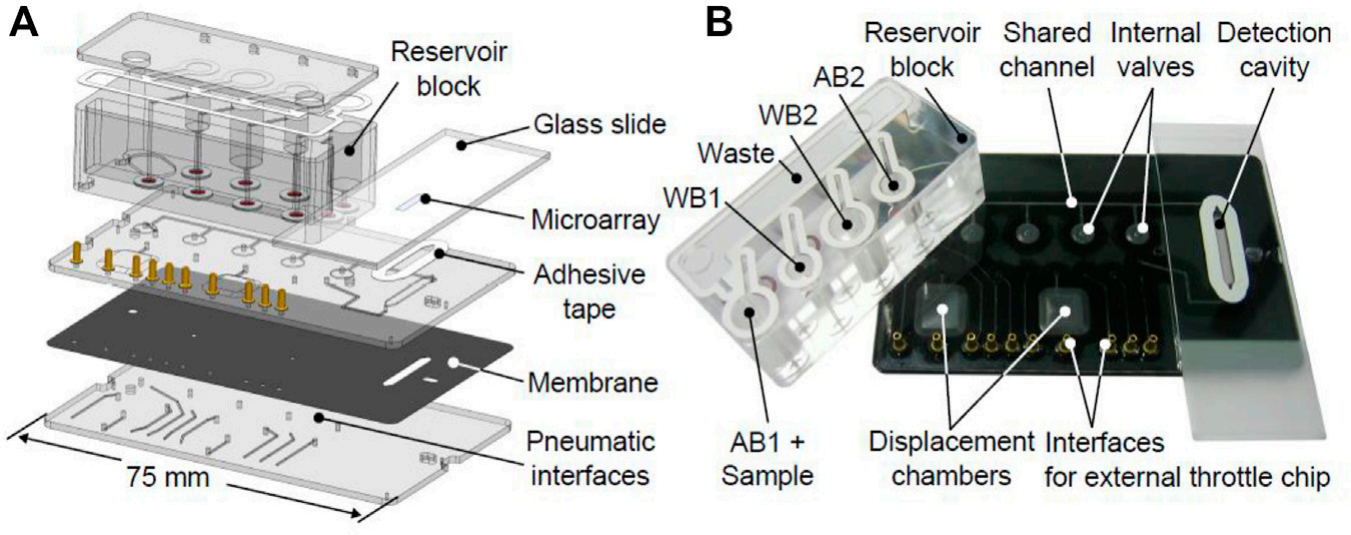
Microfluidic system developed for immunoassay experiments. **(A)** CAD drawing showing polymer with different layers constructed. **(B)** Photograph of a fabricated system.

Figure 3A) shows a CAD drawing and (b) a photograph of the microfluidic system developed based on the scheme from Figure 2. Pneumatic interface is showed at the bottom part of the system, contained to connect external pressure. The external throttle chip was routed using an interface for some of the pneumatic connections. In order to perform an easy removal of the printed glass slide after processing, a double-side adhesive tape was used to reversibly bond against the microfluidic chip. The adhesive tape was laser-cut to form an elongated channel and optimized to allow successive filling of the detection cavity with liquids and air without the risk of trapping bubbles or drops. After processing, the glass slide with the microarray could be removed and read out using a commercial reader. This approach was judged as most effective for the prototyping stage, as no separate reader had to be developed. In a final POC system, the microarray could be bonded onto the microfluidic chip irreversibly and read out using an integrated, miniaturized fluorescence reader. In order to evaluate the functionality of the microfluidic system, the inlet, outlet, and flow were assessed using colored liquids (see Supplementary Video). The assay run automatically and includes the addition of AB1+samples, WB1, AB2, WB2, and dry. According to these considerations, the printed microarray was held in the microfluidic system, and the assay was performed automatically using the protocol described in Tables 2, 3. After the drying step, the microarray was removed for the acquisition of the images.

**FIGURE 4:**
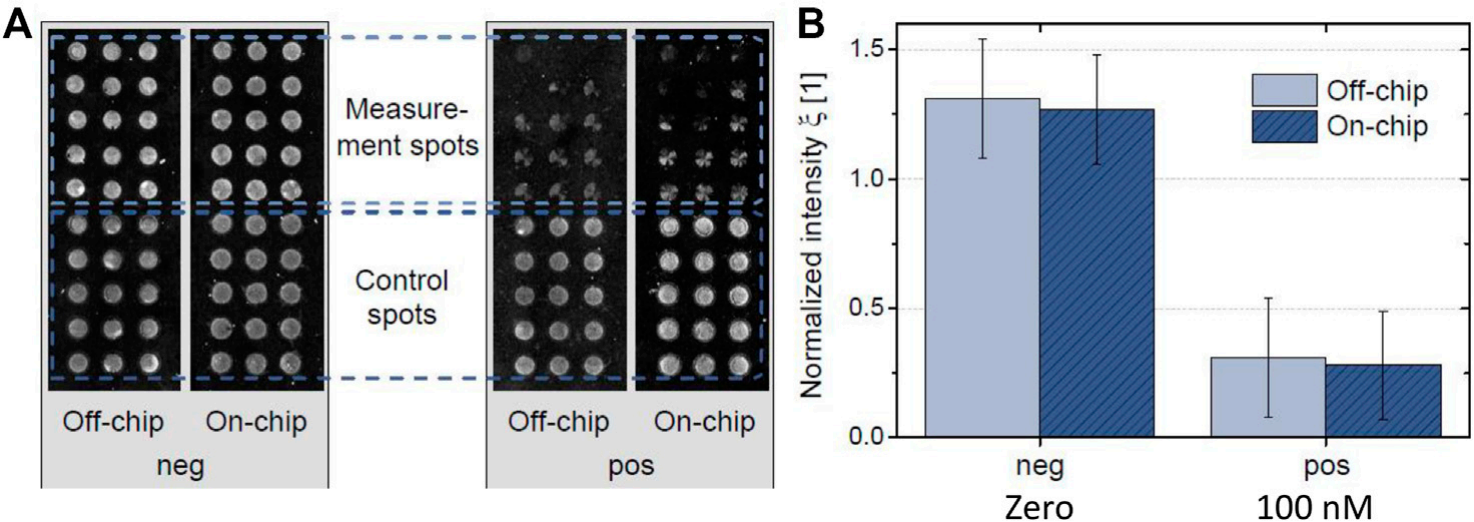
Microarray response for non-spiked and 100 nM ACL spiked plasma samples using an automatic microfluidic system (on-chip) and manually static assay (off-chip). **(A)** Fluorescent readout images showing microarrays of non-spiked and 100 nM spiked sample in on-chip and off-chip assay. The arrays were separated into control (13BSA/As138 assay) and measurement (A2BSA/As233 assay) spots, in which the latest are sensitive to the analyte. **(B)** Normalized intensity for measurements without (negative that corresponds to non-spiked sample, zero) and with analyte (positive, sample spiked at 100 nM of ACL).

Figure 4A shows the images of fully processed microarrays obtained for off-chip references and on-chip measurements. The spotted microarray was designed into two blocks, upper part corresponds to the ACL sensitive array (spotted A2BSA conjugate) and the lower part takes place the binding of the control signal (spotted 13BSA conjugate). It is expected that the upper part showed the ACL signaling and the lower part will give signals independent of the analyte concentration to confirm that the biochemical reactions during the assay were successfully performed. Two samples were tested, a blank sample (negative) and 100 nM ACL (positive), which is a concentration that can be found in the bloodstream in OAT patients. The experiment was carried out using the microfluidic system (on-chip experiment), and the assay was performed under static conditions (off-chip experiment). As can be seen from the images, a good agreement was achieved between on- and off-chip experiments. Non-specific binding was not observed from all immunoreagents used, therefore no additional protein blocking steps were performed prior to the experiments. The intensity of the control spots for each experiment was used to normalize the ACL array signal. According to the mean measurement spot intensity of both arrays, the results revealed a comparable quantitative result between them. As can be seen from Figure 4B, the signal of the non-spiked plasma sample (negative) was significantly different compared to the 100 nM spiked sample one both for on- and off-chip experiments.

Thus, these experiments showed that the fluidic workflow for an automated immunoassay was successfully carried out within an integrated format. The immunoassay for ACL determination was carried out using the pre-stored fluids in the external reservoir, which were successfully supplied and routed toward the detection cavity. Moreover, a good agreement was found between on-chip measurements and reference was obtained, meaning that no significant cross-contamination between the different reagents took place.

## Conclusions

A highly sensitive and specific fluorescent microarray assay was developed for the detection of ACL, an important drug used as OAT. The assay developed maintains the analytical features set previously by ELISA and showed the potential use for the direct detection of ACL in plasma. The aim of this assay was to implement it in a microfluidic device for the easy and automatically development of a point-of-care device that helps the clinicians take decisions based on the concentration found. To do that, the assay was optimized to be used in a minimum time of assay and using an internal standard to improve the reproducibility of the assay.

On the other hand, we have demonstrated proof-of-principle for the implementation of an immunochemical assay for the detection of oral anticoagulants in an automated microfluidic system. A technology for precisely positioning aliquots of liquid above a detection area has been developed. The developed microfluidic system implements the two unit functions “position above detection area” and “flush detection area” and could be modified for realizing arbitrary immunochemical assays very easily in the sense of a processing platform also for other applications.

## Data Availability

The raw data supporting the conclusions of this article will be made available by the authors, without undue reservation.
